# Potential of Sentinel-1 Surface Soil Moisture Product for Detecting Heavy Rainfall in the South of France

**DOI:** 10.3390/s19040802

**Published:** 2019-02-16

**Authors:** Hassan Bazzi, Nicolas Baghdadi, Mohammad El Hajj, Mehrez Zribi

**Affiliations:** 1 IRSTEA, TETIS, University of Montpellier, 500 rue François Breton, 34093 Montpellier CEDEX 5, France; nicolas.baghdadi@teledetection.fr (N.B.); mohammad.el-hajj@teledetection.fr (M.E.H.); 2 CNRS/UPS/IRD/CNES, CESBIO, 18 av. Edouard Belin, bpi 2801, 31401 Toulouse CEDEX 9, France; mehrez.zribi@ird.fr

**Keywords:** Sentinel-1, global precipitation measurement, soil moisture, heavy rainfall

## Abstract

The objective of this paper is to present an analysis of Sentinel-1 derived surface soil moisture maps (S1-SSM) produced with high spatial resolution (at plot scale) and a revisit time of six days for the Occitanie region located in the South of France as a function of precipitation data, in order to investigate the potential of S1-SSM maps for detecting heavy rainfalls. First, the correlation between S1-SSM maps and rainfall maps provided by the Global Precipitation Mission (GPM) was investigated. Then, we analyzed the effect of the S1-SSM temporal resolution on detecting heavy rainfall events and the impact of these events on S1-SSM values as a function of the number of days that separated the heavy rainfall and the S1 acquisition date (cumulative rainfall more than 60 mm in 24 hours or 80 mm in 48 hours). The results showed that the six-day temporal resolution of the S1-SSM map doesn’t always permit the detection of an extreme rainfall event, because confusion will appear between high S1-SSM values due to extreme rainfall events occurring six days before the acquisition of S1-SSM, and high S1-SSM values due to light rain a few hours before the acquisition of Sentinel-1 images. Moreover, the monitoring of extreme rain events using only soil moisture maps remains difficult, since many environmental parameters could affect the value of SSM, and synthetic aperture radar (SAR) doesn’t allow the estimation of very high soil moistures (higher than 35 vol.%).

## 1. Introduction

Understanding the Earth’s global water cycle requires the spatiotemporal monitoring of precipitation [1]. Moreover, precipitation monitoring is essential, since rainfall directly affects human life. Indeed, very high rainfall episodes may cause runoff, which damages life and property, whereas the absence of precipitation can produce droughts [2]. Thus, knowing the extent of rain helps us better understand the effect of climate and weather on agriculture and water availability. 

The registration of in situ rain gauges allows only the measurement of local precipitation amounts. Thus, these rain gauge instruments are not sufficient to provide robust continuous mapping of rainfall, because rainfall events can be very local. Moreover, in situ rain gauge registrations are not usually freely available. On the other hand, remote sensing metrological sensors enable the continuous mapping of precipitation in open access mode. Currently, several metrological sensors provide rainfall mapping with a high revisit time and low spatial resolution: the Tropical Rainfall Measuring Mission (TRMM) (resolution ~0.25°×0.25°) [3], the Precipitation Estimation from Remotely Sensed Information using Artificial Neural Network (PERSIANN) (resolution ~0.25°×0.25°) [4], the Climate Prediction Center Morphing (CMORPH) (resolution ~8 km × 8 km at the equator) [5]. The most recent sensor, Global Precipitation Measurement (GPM), provides precipitation mapping at 0.1°×0.1° spatial resolution and a high revisit time (up to 30 minutes) [6]. The low spatial resolution makes the available metrological sensors not adequate for hydrological application, flood forecasting, and water resource management. For example, the spatial detection of extreme rainfall events remains difficult due to the low spatial resolution of available precipitation data. Extreme rainfall events can be defined as rainy episodes with high cumulative precipitation in short periods (24 to 48 hours), which is equal to that usually received in one month. 

An important variable affected by precipitation events is the surface soil moisture. In fact, surface soil moisture can be a key tool for detecting rain events, because soil moisture is a function of the precipitation rate, evaporation, runoff, irrigation, and snow melt. Eventually, an increase in the soil moisture values could be primarily associated with a precipitation event, assuming that no irrigation activity is performed. Currently, several satellite missions provide surface soil moisture estimations at different spatial resolutions such as the Soil Moisture Active Passive “SMAP” (level 3: 36 km × 36 km, level 3 enhanced: 9 km × 9 km, and Level 2 SMAP/Sentinel-1: 1 km × 1 km) [7], the Advanced Scatterometer “ASCAT” (level 2 with three spatial resolutions: 25 km × 25 km, 12.5 km × 12.5 km, and 1 km × 1 km) [8], and the Soil Moisture and Ocean Salinity “SMOS” (SMOS INRA-CESBIO level 3: 25 km × 25 km) [9], the Advanced Microwave Scanning Radiometer 2 “AMSR2” [10] (with two spatial resolutions 0.1°×0.1° and 0.25°×0.25°), and the Chinese Fengyun-3B “FY3B” (spatial resolution of 0.25°×0.25°) [11].

Using soil moisture information, Pellarin et al. [12] proposed a simple method to correct the TRMM and PERSIANN precipitation estimations at low spatial resolution (~0.25°×0.25°). However, in the last three decades, numerous approaches have been developed to retrieve soil surface parameters using synthetic aperture radar (SAR) remote sensing [13,14,15]. From soil surface parameters, Sentinel-1 gives soil moisture estimations at very high spatial resolution (plot scale). In fact, with the arrival of Sentinel-1 (S1 SAR data at 10-m spatial resolution and a six-day revisit period) and Sentinel-2 (S2 optical data at 10-m spatial resolution and a five-day revisit period) satellites, an operational algorithm has been developed for soil moisture mapping over agricultural areas with six days’ revisit time and high spatial resolutions (up to plot scale) [16]. 

The objective of this paper is to analyze the variation in surface soil moisture values obtained from the very high spatial resolution Sentinel-1 surface soil moisture maps (S1-SSM) during one year over the Occitanie region in the south of France. In this study, the variation of the SSM is analyzed only according to the variation of rainfall derived from GPM data. Although the water cycle components, such as evaporation rate and snow melt, play a critical role in SSM variation, these components were not considered in this study. This study will allow us to investigate the opportunity to detect heavy rainfall using Sentinel-1 surface soil moisture products obtained with a revisit time of six days at high spatial resolution in the south of France. After a detailed description of the study site and the data used in Section 2, Section 3 includes a comparison between S1-SSM and precipitation, as well as an analysis of the possibility of detecting heavy rainfall using S1-SSM with a revisit time of six days in the south of France (Occitanie region). A discussion is presented in Section 4, including the effect of S1 temporal resolution on detecting heavy rainfall and investigating the potential of SMAP soil moisture product with a revisit time of one day to detect heavy rainfall. Finally, Section 5 presents the main conclusions.

## 2. Study Site and Dataset Description

### 2.1. Study Site

**Figure 1:**
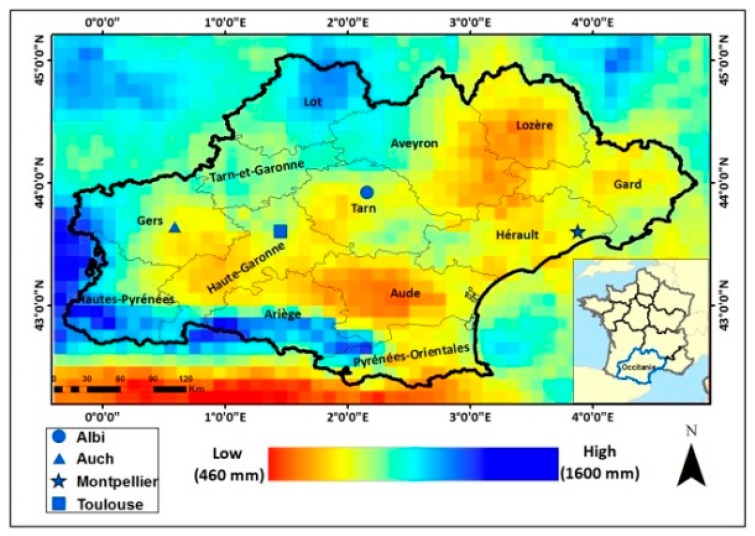
Annual cumulative precipitation calculated using Integrated Multi-satellite Retrievals for Global Precipitation Measurement (IMERG-GPM) data over the study site (Occitanie region, South France) between 1 September 2016 and 31 August 2017. Black lines represent the department limits according to French administrative nomenclature.

The study site is the Occitanie region in the south of France (centered on $2{}^{\circ}30^{\prime }\;E\;\mathrm{and}\;43{}^{\circ}30^{\prime }\;N$, Figure 1) covering an area of 72724 km^2^. The region is formed out of 13 departments (Figure 1). Having a rich variety of landscapes, Occitanie is mainly covered by agricultural areas (especially wheat and corn) in the western part with a mix of crops, mountains, and forests in the eastern and southern parts. The region comprises different climatic zones. The eastern part is considered Mediterranean (about 700 mm of average annual precipitation in Hérault, Figure 1), while the western part is more humid (about 1200 mm of average annual precipitation in Gers, Figure 1).

### 2.2. Dataset Description

#### 2.2.1. S1-Derived Soil Moisture Maps

Sentinel-1 soil moisture maps (S1-SSM) at very high spatial resolution (up to a plot scale with a minimum area of 0.2 hectares) over our study area were analyzed. These soil moisture maps were operationally produced by coupling SAR data from both the S1A and S1B satellite constellations operating at the C-band (wavelength ~6 cm) and optical data from both the S2A and S2B satellite constellations [16]. To estimate the soil moisture over agricultural areas, El Hajj et al. [16] inverted the Water Cloud Model (WCM) [17] combined with the Integral Equation Model (IEM) [18]. In their study, they used the WCM parameterized by Baghdadi et al. [19] for the C-band. The total backscattering coefficient in the WCM is considered the sum of the direct vegetation contribution and the soil contribution multiplied by the attenuation factor. Using a vegetation descriptor derived from optical images (Sentinel-2), the direct contribution of vegetation can be calculated. El Hajj et al. [16] validated the proposed approach for the operational mapping of soil moisture over a study site in Occitanie in South France. They showed that the soil moisture in agricultural areas could be estimated with an accuracy of approximately 5 vol.%. Moreover, El Hajj et al. evaluated SMOS, SMAP, ASCAT, and S1-SSM products [20], and revealed that the S1-derived soil moisture maps provide the most accurate estimation of SSM. The higher accuracy of estimated SSM moisture is probably due to the well-calibrated IEM combined with the well-parameterized WCM and the use of high spatial resolution (10 m×10 m) land cover maps derived from S2 images to eliminate SAR scattering from forest and urban areas. S1-SSM maps are produced for agricultural areas masking forest, urban, and high-slope areas (slope >20% calculated based on elevation data provided by the Shuttle Radar Topography Mission “SRTM” at 30 m × 30 m spatial resolution). S1 soil moisture maps for the Occitanie region are available as open access data via the Theia French Land Data Center (http://www.theia-land.fr/en/thematic-products). The SSM map at each date was generated using the ascending acquisition mode of the Sentinel-1 images (acquisition time ~17:40 TU). Four maps are required to cover the region on a single date. Thus, a total of 250 soil moisture maps were downloaded to cover the whole region on the studied dates.

#### 2.2.2. IMERG GPM Products

Rainfall measurements with 30-minute revisit time that were obtained from the GPM (Global Precipitation Measurements) sensor were considered in this study. The GPM mission is an international satellite mission initiated by the National Aeronautics and Space Administration (NASA) and the Japan Aerospace and Exploration Agency (JAXA) to unify and advance global precipitation measurements from space [6]. GPM provides several precipitation records at different scales by using different sensor combinations. From the GPM mission, the IMERG (Integrated Multi-satellite Retrievals for GPM) data product was selected to perform our analysis. IMERG data offer global precipitation estimations in both real-time and late mode. While real-time IMERG data are proposed for disaster monitoring and flood risk assessment, the higher quality late IMERG data are intended for meteorological and climatological applications. The spatial resolution of IMERG maps is 0.1°×0.1°, which fully covers the globe between 60° N and 60° S. These data provide precipitation maps with several time intervals (30 minutes, 24 hours, three days…). The NASA GPM program established several campaigns to collect ground-based observations to validate GPM products [21]. Omranian et al. [22] validated the accuracy of GPM-IMERG data over the Colorado River basin in Texas, and found that the GPM-IMREG slightly underestimates the precipitation over the basin. Several studies have also reported good correlation between GPM-IMREG products and rain gauges with small under or overestimation depending on the study site, and better estimation when compared to other precipitation products [23]. IMERG data at different spatial and temporal resolutions are available via the NASA Precipitation Measurements Mission (PMM) website (https://pmm.nasa.gov/data-access). In this study, the fifth version (V05) of GPM-IMERG products was used.

## 3. Data Analysis

### 3.1. Comparison between S1-SSM and Precipitation

**Figure 2:**
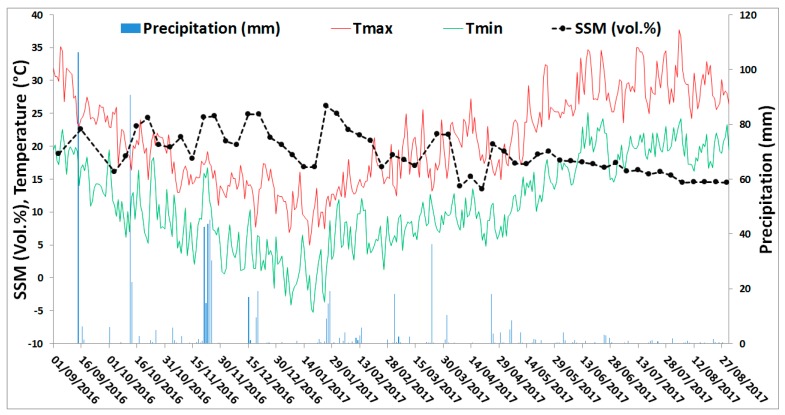
Temporal evolution of soil moisture values of Sentinel-1 derived surface soil moisture maps (S1-SSM) with daily precipitation records for a GPM grid cell over Montpellier (0.1°×0.1°), and daily temperature records (minimum and maximum temperature) at a local station in Montpellier, Occitanie region, France.

A first analysis of soil moisture variation according to precipitation events was performed. For a grid cell of the GPM product (0.1°×0.1°) over Montpellier (Figure 1), GPM daily cumulative precipitation records were obtained over one year between September 2016 and August 2017. Then, at each available S1-SSM map between 1 September 2016 and 31 August 2017, mean soil moisture values were calculated for the corresponding GPM grid cell. Figure 2 shows the temporal variation in the mean soil moisture values, the daily precipitation records, and the daily air temperature records (minimum and maximum daily air temperature). A global consistency is observed between the S1-SSM values and precipitation records. Figure 2 shows that after a precipitation event, high soil moisture values are estimated, whereas with the absence of precipitation over a period of time, the soil dries due to evaporation, and S1-SSM values generally decrease. For example, an increase in soil moisture values from 16 vol.% on 15 November 2016 to 25 vol.% on 21 November 2016 was observed due to 40-mm precipitation recorded on 20 November 2016. Also, soil moisture values increased from 19 vol.% on 9 December 2016 to 25 vol.% on 15 December 2016 due to 17 mm of precipitation recorded on 15 December 2016. Moreover, the absence of precipitation along with evaporation (maximum temperature between 22 °C and 25 °C) between 1 April 2017 and 25 April 2017 caused soil moisture values to drop down below 15 vol.%, followed then by a sharp increase in soil moisture values on 26 April 2017 (24 vol.%) due to rainfall event occurring on 25 April 2017 (13 mm). On the other hand, 18 mm of precipitation on 4 March 2017 did not highly affect the soil moisture values on 9 March 2017 (18 vol.%) due to the five-day difference between the rainfall date and the following S1-SSM date. For the period between May and August, the increase of temperature (and consequently the evaporation) didn’t cause a sharp decrease in SSM values. This is probably due to the existence of irrigation activities for summer crops. According to the six-day revisit time of the Sentinel-1 sensor, it is difficult to quantitatively analyze the correlation between the precipitation and the soil moisture, because this correlation also depends on other parameters such as the evaporation rate, the soil texture, and the previous state of soil before rainfall events. For this reason, the correlation coefficient R was only calculated between the average annual soil moisture for each department and the corresponding annual precipitation (Figure 1). A correlation coefficient R of 0.89 was obtained between the mean annual soil moisture at each department and the average annual cumulative precipitation of each department.

The temporal analysis of the S1-SSM values along with GPM data was then studied during the period between 21 December 2016 and 1 February 2017. Different climatological events were observed during this period: the absence of precipitation for 24 consecutive days in some parts of the region between 27 December 2016 and 25 January 2017 and rain episodes at several dates (Figure 2). The objective here is to analyze the temporal evolution of S1-derived SSM as a function of cumulative rainfall events (existence or absence of precipitation). 

As mentioned in Section 2.2.1, an S1 soil moisture map can be obtained every six days (during the revisit of the S1 satellite). Thus, a total of eight soil moisture maps acquired between 21 December 2016 and 1 February 2017 were used. For these maps, soil moisture values were classified into four different intervals that were found to best describe the spatiotemporal variation in soil moisture values. 

**Figure 3:**
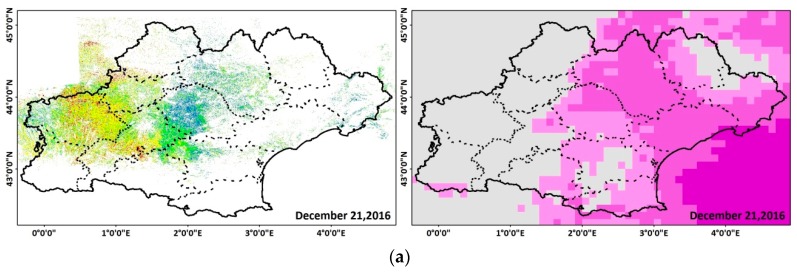

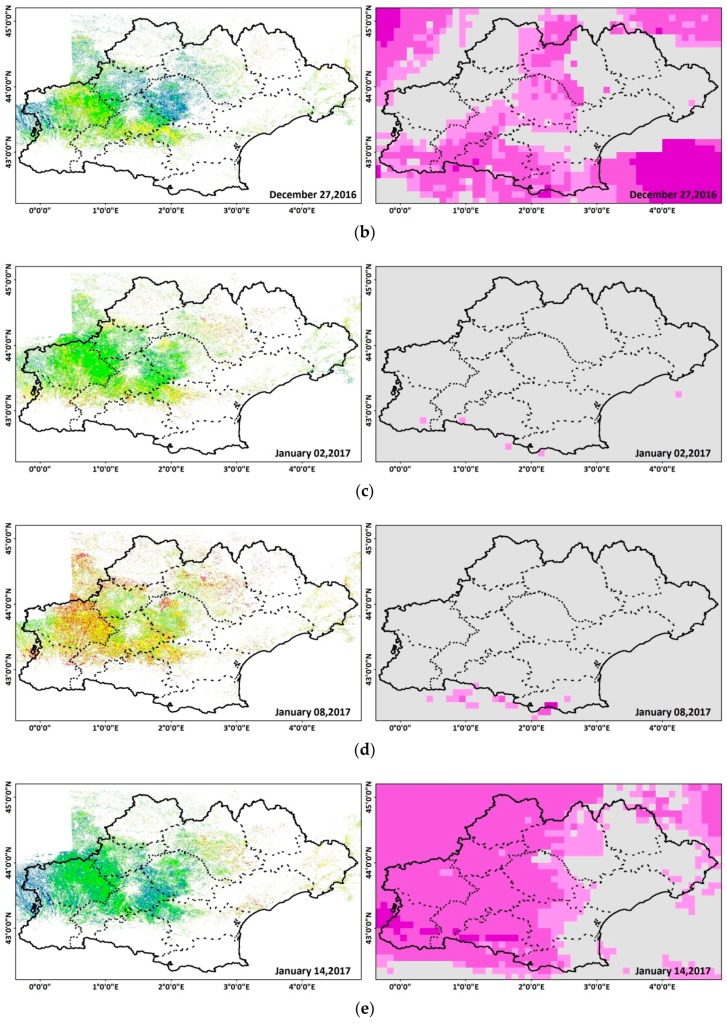

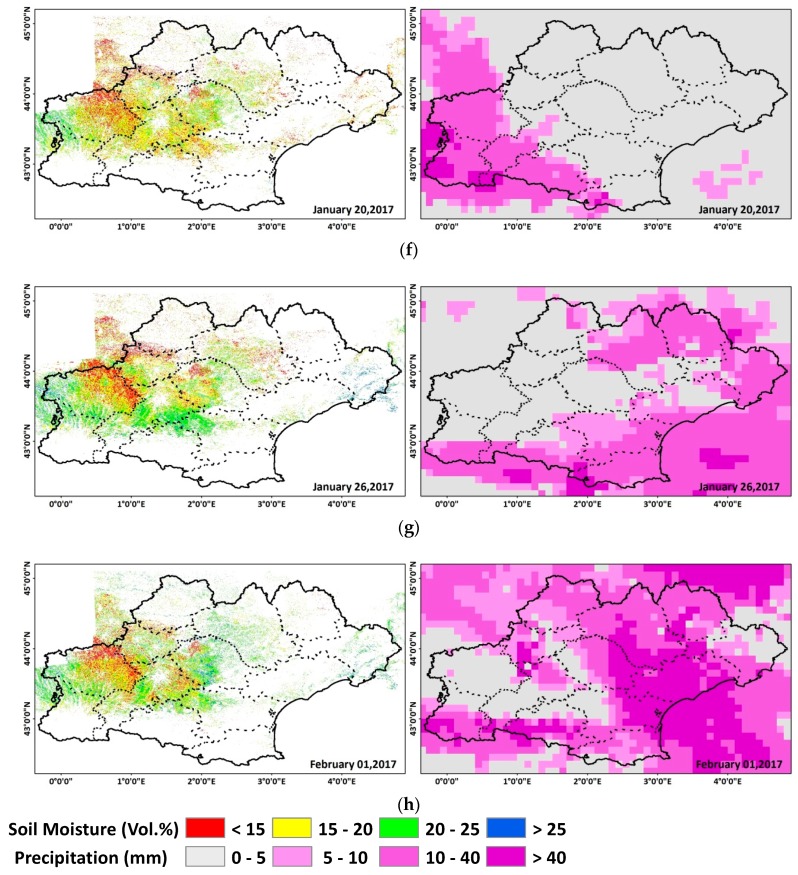
On the left, an S1-derived SSM map. On the right, the corresponding six days of GPM cumulative precipitation data. (**a**) 21 December 2016, (**b**) 27 December 2016, (**c**) 2 January 2017, (**d**) 8 January 2017, (**e**) 14 January 2017, (**f**) 20 January 2017, (**g**) 26 January 2017, and (**h**) 1 February 2017. Departments are represented by dashed lines.

To perform the temporal evolution analysis, first, for each S1-SSM map date, one cumulative rainfall map for the six days prior to the S1-SSM map date was computed by summing the 30-minute time interval of the late IMERG-GPM product. Then, each S1-derived SSM map was overlaid with the corresponding six days of cumulative GPM rainfall. From the qualitative analysis of the overlaid maps, several arguments can be extracted and discussed. Starting with the initial map on 21 December 2016 (Figure 3a), departments as Tarn, Hérault, Lozère, and Aveyron of the eastern and middle parts of the region encountered high soil moisture values (more than 20 vol.%) corresponding to 10 to 20 mm of cumulative precipitation during the past six days, whereas Gers and Haute-Garonne in the western part showed low soil moisture values (20 vol.% at most) corresponding to the absence of precipitation in the area for the same prior period. A map derived six days later on 27 December 2016 (Figure 3b) shows a decrease in soil moisture values in the Hérault and Gard regions due to the absence of precipitation between 21 December 2016 and 27 December 2016. Moreover, high soil moisture values were estimated for Tarn due to continuous raining episodes between 21 December 2017 and 27 December 2017, and a slight increase in soil moisture values was observed in western part due to three mm of precipitation recorded one day prior to the S1-SSM map, according to the GPM data (Figure 3b). A general decrease in the soil moisture values throughout the region was observed between 27 December 2016 (Figure 3b) and 2 January 2017 (Figure 3c), as no precipitation was recorded. A continuous lack of precipitation for 12 days from 27 December 2016 until 8 January 2017 caused soil moisture values to drop to less than 15 vol.% on 8 January 2017 in the whole region, as shown in Figure 3d. Six days later on 14 January 2017 (Figure 3e), soil moisture values remained low at the east, as no precipitation was recorded for an additional six days, whereas a moderate increase in soil moisture values was observed in the middle and western parts (Tarn, Haute-Garonne, and Gers), corresponding to precipitation between 8 January 2017 and 14 January 2017 (a cumulative rainfall increase of five to 20 mm). Twenty-four days with no precipitation recorded in the Hérault and Gard region between 27 December 2016 and 20 January 2017 caused the soil moisture values to drop more, with values less than 15 vol.% in this area on 20 January 2017 (Figure 3f). 

**Figure 4:**
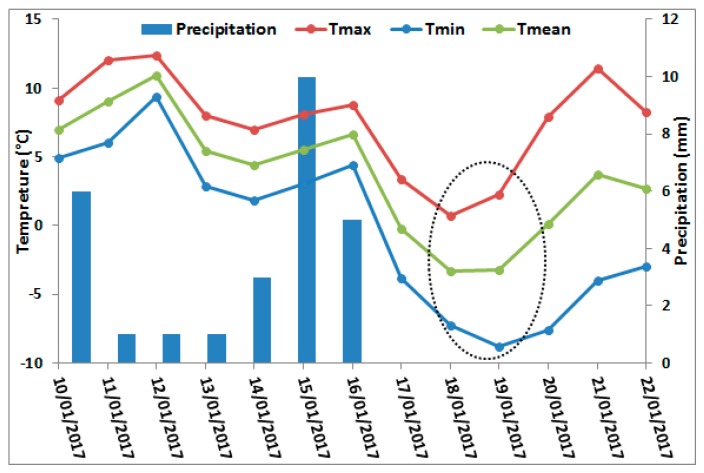
Temporal evolution of temperature and precipitation data recorded at a local meteorological station in Auch, Occitanie region.

In addition, for the Gers department, low soil moisture values (less than 15 vol.%) were estimated on 20 January 2017 and high cumulative precipitation was recorded between 14 and 20 January 2017 (15 mm) (Figure 3f). This low soil moisture estimates could be linked to the presence of frozen soil in the area. In a recent study using Sentinel-1 SAR data, Baghdadi et al. [24] showed that a decrease of at least three dB in the radar backscattered signal can be observed over frozen soil conditions. Such a decrease in the SAR signal yields an underestimation of soil moisture in the S1-derived SSM maps. In our case, it seems that the low soil moisture values for this area on 20 January 2017, although with the presence of rainfall, were due to frozen soil conditions. To support our assumption, the temperature curve and precipitation records for a local meteorological station in Auch city located in Gers-Occitanie were analyzed (Figure 4). On 19 January 2017 (S1 acquisition over Gers on 19 January 2017 17:56 UTC), the temperature varied between –8 °C and 2 °C with a mean value of –3 °C throughout the day. Three days before (15 and 16 January), 15 mm of cumulative precipitation was recorded. Thus, a precipitation event followed by a decrease in the air temperature to less than 0 °C justifies the presence of frozen soil conditions as the cause of a decrease in the estimated soil moisture values in the S1-derived SSM maps.

After 29 days of dry conditions in the eastern part (Hérault and Gard between 27 December 2016 and 25 January 2017), a sudden increase in the soil moisture was observed on 26 January 2017 with the beginning of a rainfall event (Figure 3g). The mean S1-SSM value in this area increased from 13 vol.% on 20 January 2017 to 32 vol.% on 26 January 2017. On the other hand, the absence of precipitation in the remaining parts of the region between 20 January 2017 and 26 January 2017 caused the soil moisture to attain lower values (16 vol.%) on 26 January 2017. The slight increase in soil moisture values in Gers between 20 and 26 January is probably due to the disappearance of the frozen conditions observed in the previous acquisition (20 January 2017). The map on 1 February 2017 (Figure 3h) shows high soil moisture values for the eastern part, which was affected by continuous raining events, and a general decrease in the western part due to the absence of rainfall between 20 January 2017 and 1 February 2017. The temporal analysis of S1-derived SSM values over a period of time shows the direct effect of raining episodes or dry conditions on S1-SSM values. 

### 3.2. Effect of S1-SSM Temporal Resolution

**Figure 5:**
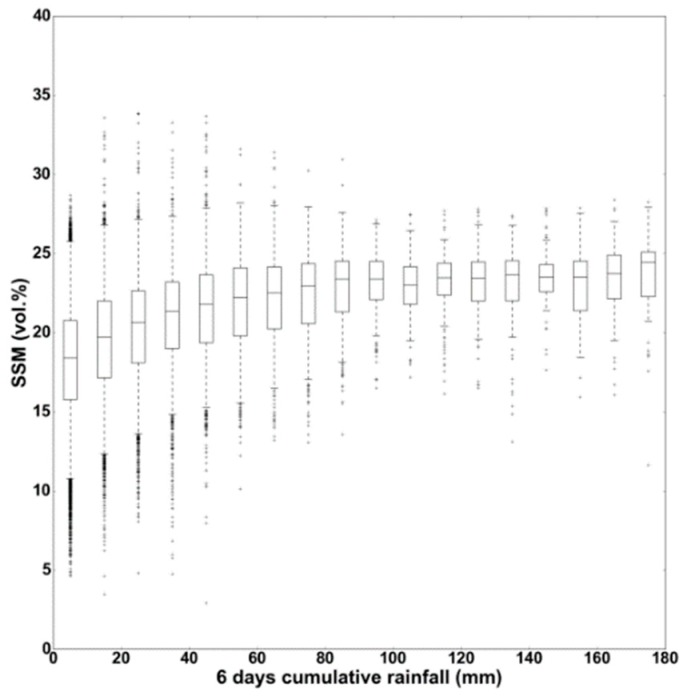
S1-SSM distribution as a function of six days of cumulative precipitation (revisit time of S1-SSM = six days).

The behavior of soil moisture values following rainfall events was then studied as a function of the time lag between the rainfall date and the S1-SSM date. In fact, soil moisture values are mainly affected by precipitation events, evaporation, runoff, and snow melt. In this analysis, only the effect of precipitation was studied. To study the effect of the time difference between the date of S1-SSM maps and the date of rainfall events, S1-SSM values were plotted as a function of the recorded six days of cumulative precipitation values (Figure 5). For the 0.1°×0.1° GPM grid, the mean S1-SSM values were calculated from each available S1-SSM map. The cumulative precipitation was computed for six days, because the revisit time of derived S1-SSM maps is for six days over our study site. The results showed that for low cumulative precipitation for six days preceding the S1-SSM map date (for example, less than 20 mm), the soil moisture values are distributed on a wide range between 10–25 vol.% (Figure 5). This wide range of S1-SSM values was expected, since low precipitation would induce high S1-SSM values if the precipitation occurs in the 24 hours preceding S1 acquisition, whereas low precipitation would not induce an increase of S1-SSM if it occurs a couple of days before S1 acquisition. As the cumulative precipitation for six days is high (for example 100 mm, Figure 5), the S1-SSM values become more homogenous and high. Indeed, high cumulative precipitation for six days (for example, higher than 100 mm) induces high S1-SSM values, even if the precipitation occurs six days before S1 acquisition, in particular during winter, and the evaporation rate is low.

**Figure 6:**
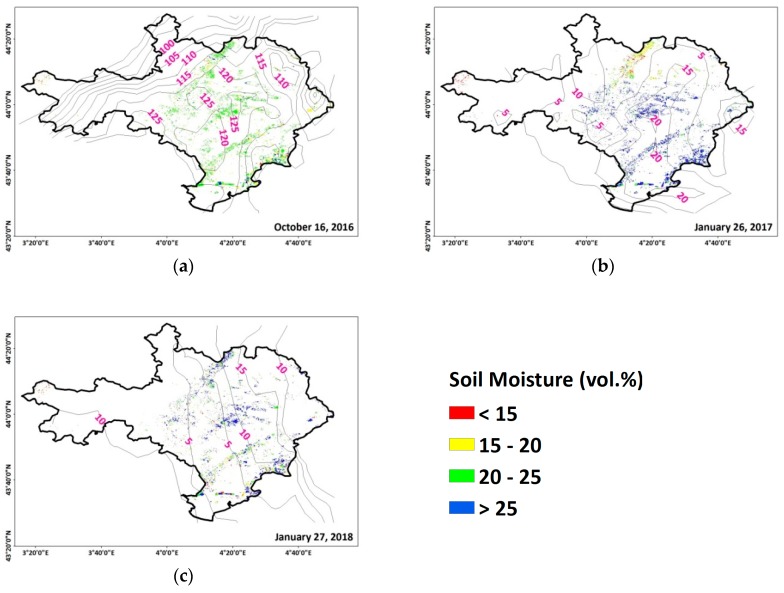
S1-derived SSM maps overlaid with GPM precipitation data. (**a**) 16 October 2016, (**b**) 26 January 2017, and (**c**) 27 January 2018. Contour lines represent precipitation amounts in mm.

To illustrate the results presented in Figure 5, three cases of rainfall events with different time intervals between the S1-SSM map’s date and the rainfall date were studied for Gard Department of Occitanie. First, for the S1-SSM map of 16 October 2016, the latest rainfall event before 16 October 2016 took place on 12 October 2016 with an average cumulative precipitation of 100 mm in 24 hours (Figure 6a.) Then, between 12 October 2016 and 16 October 2016, no precipitation was recorded. The soil moisture map of 16 October 2017 shows wet soil conditions with S1-SSM values between 20–25 vol.%. On the other S1-SSM map acquired on 26 January 2017, a rainfall event occurred on the same date a few hours before the S1 acquisition date (26 January 2017) with 20 mm of cumulative precipitation (Figure 6b). Consequently, the soil moisture attained very high values: higher than 25 vol.%. The department didn’t encounter any rainfall event for 29 days prior to 26 January 2017 (the soil moisture map that was acquired six days before 26 January 2017 showed soil moisture values of 13 vol.%). Similar soil moisture values were recorded on the S1-SSM map of 27 January 2018 (Figure 6c), where the department was affected by a light rain event 24 hours before the S1-SSM date (nine mm). Wet soil moisture values higher than 25 vol.% were estimated over the department. Prior to this rainfall event on 26 January 2018, no events were detected for the previous six days, and the soil moisture values six days before presented dry values (15 vol.%). This result assures that the detection of rainfall events on S1-SSM is highly dependent on the S1-SSM revisit time. Indeed, a few mm of precipitation just 24 hours before the S1-SSM map shows higher soil moisture values (for example on 27 January 2018) than an extreme event rainfall event (for example on 12 October 2016) four days before the S1-SSM map (16 October 2016). Thus, the six days of temporal resolution of the S1-SSM map doesn’t always permit the detection of an extreme rainfall event. For example, confusion will appear between high values due to extreme rainfall events occurring six days before the S1-SSM acquisition and high values due to light or moderate rain a few hours before the S1 acquisition date.

## 4. Discussion

The results obtained in Section 3 show that the detection of heavy rainfall using S1-SSM values is highly dependent on the time lag between the heavy rainfall event and the S1-SSM acquisition. Figure 5 showed that, on average, SSM values varied between 15 vol.% for five mm of accumulated precipitation in six days to about 22 vol.% for accumulated precipitation greater than 100 mm in six days. In addition, when the accumulated precipitation was less than 30 mm, 75% of the pixels (0.1° × 0.1°) showed SSM values lower than 22 vol.%, and the remaining 25% had SSM values between 20–28 vol.%. On the other hand, when the accumulated precipitation is higher than 100 mm in six days, 75% of the pixels have SSM values higher than 22 vol.%, while the remaining 25% of the pixels have SSM values between 20–22 vol.%. Moreover, for six days of cumulative precipitation between 30–100 mm, the SSM values varied between 15–28 vol.%. Thus, the results show that low rainfall of less than 30 mm in six days and very heavy rainfall greater than 100 mm in six days generally result in different soil moisture conditions in approximately 75% of the cases. However, it remains difficult to distinguish between high SSM values (around 28 vol.%) corresponding to (1) light rain the day before the S1 acquisition, (2) heavy rainfall a few days before the radar acquisition, or (3) various light rainy episodes during several days preceding the radar acquisition. Therefore, the SSM maps with a revisit period of six days do not always allow us to discriminate wet soil following a light rain at a date very close to the date of acquisition of the S1 image, and wet soil following a heavy rain that took place several days before the S1 acquisition date.

**Figure 7:**
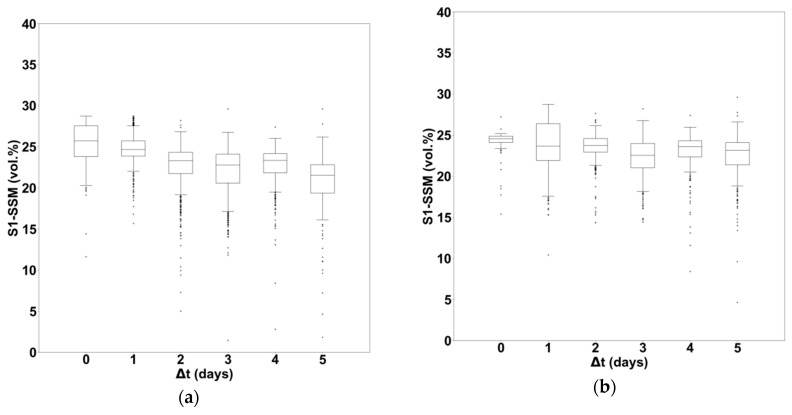
S1-SSM distribution as a function of the number of days that separate the heavy rainfall event and the acquisition date of S1. (**a**) Heavy rainfall with 60 mm of cumulative precipitation in 24 hours, (**b**) heavy rainfall with 80 mm of cumulative precipitation in 48 hours.

Moreover, we have analyzed the impact of heavy rainfall (cumulative rainfall more than 60 mm in 24 hours or more than 80 mm in 48 hours) on S1-SSM values as a function of the number of days that separated the heavy rainfall event and the S1 acquisition date ($∆t=t_{S1}-t_{Heavy\;Rainfall})$. These threshold values of 60 mm and 80 mm were selected according to Meteo-France recommendations (http://pluiesextremes.meteo.fr/france-metropole/Statistiques-et-records.html). Figure 7 shows the SSM values as a function of ∆t for cumulative rainfall greater than 60 mm in 24 hours (Figure 7a) and 80 mm in 48 hours (Figure 7b). For example, ∆t=0 corresponds to a heavy rainfall event that occurred during the past 24 hours (or 48 hours) of the S1 acquisition. In the case of a cumulative rainfall greater than 60 mm in 24 hours (Figure 7a), we find that the SSM average values decreases from 25.4 vol.% for ∆t=0 to 20.8 vol.% for ∆t=5 days (S1 image acquired six days after the heavy rainfall event). In the case where the cumulative rainfall is greater than 80 mm in 48 hours (Figure 7b), we find that SSM average values are similar (23 vol.%) after a heavy rainfall that took place during the past 48 hours of the S1 acquisition or after a heavy rainfall during the 48 hours that took place six days before the S1 acquisition.

**Figure 8:**
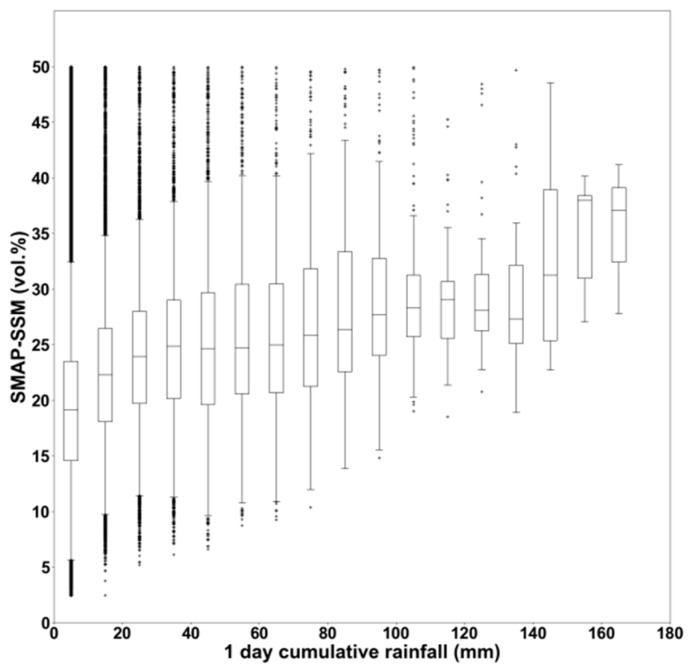
Soil Moisture Active Passive (SMAP)-SSM distribution as a function of one day of cumulative precipitation (revisit time of SMAP-SSM = one day).

The effect of the temporal resolution of satellite images on detecting heavy rainfall events was further studied with soil moisture data from SMAP at higher temporal resolution [7]. For this reason, we have re-established Figure 5 with SMAP data at 9 km × 9 km spatial resolution (Figure 8). SMAP data were processed for the period between September 2016 and August 2017, over the Occitanie region, with one day of temporal resolution. Figure 8 shows the distribution of SMAP soil moisture values as a function of one day of cumulative precipitation obtained from the IMERG-GPM data. In average, SMAP shows an estimation of 15 vol.% following light rain one day before the SMAP acquisition, and about 30 vol.% following 100 mm of cumulative rainfall during one day before the SMAP acquisition. Although the difference in soil moisture between the two events (light rain and heavy rain) is greater for SMAP (about 15 vol.%) than that of S1 (7 vol.%), the results obtained with SMAP at 9 km × 9 km and one-day revisit do not also allow the easier detection of heavy rainfall events. However, the potential of the SMAP in detecting heavy rainfall appears to be better than the potential of S1 for rainfall events >150 mm, where SMAP-SSM shows values greater than 30 vol.%.

The previous discussion leads to the conclusion that soil moisture, which is obviously correlated with the amount of rainfall, depends also on other water cycle parameters such as evaporation, soil type, and the previous state of soil moisture before the heavy rainfall. Although it is obvious that heavy rainfall will lead to very wet soil, a slight rain over already wet soil will also result in extremely wet soil. A revisit time of several days (case of S1) makes the detection of heavy rainfall more complicated, because an extreme rainfall event that takes place four or five days before the S1 acquisition and is then followed by a significant evaporation will not lead to very high soil moisture values. In addition, radar sensors don’t correctly estimate soil moisture for SSM values above 30 to 35 vol.%. Baghdadi et al. [25] demonstrated that the radar signal is stable when the SSM values ranged between 30–35 vol.%, and it decreases beyond this threshold. This eventually leads to an underestimation of SSM beyond this threshold value [26]. Thus, the configurations for having very wet soil are numerous, and do not always depend on the amount of rain that falls. That many environmental parameters affect the value of SSM and the limitation of SAR to estimate the very high SSM values makes the monitoring of extreme rain events very delicate when it is based only on soil moisture maps. Therefore, the analysis carried out in this paper confirms that the use of the S1-SSM maps with a revisit period of six days (at plot scale) or the SMAP-SSM maps with very high temporal resolution of one day (at 9 km × 9 km) makes the detection of heavy rainfall not always obvious.

## 5. Conclusions

This letter presents an analysis of Sentinel-1 derived surface soil moisture maps (S1-SSM) produced with high spatial resolution (at plot scale) and a revisit time of six days for the Occitanie region located in the south of France as a function of precipitation data derived from the GPM mission, in order to investigate the potential of S1-SSM maps in detecting heavy rainfalls. First, we investigated the temporal evolution of the soil moisture according to precipitation records derived from the GPM data. A general coherence was observed between the soil moisture estimations and precipitation records over the study site. The S1-SSM values increase following rainfall events and decrease after a period without rainfall due to evaporation. Then, we studied the behavior of soil moisture values following rainfall events in order to explore the possibility of detecting heavy rainfall using the revisit time of six days of the S1-SSM product. The influence of heavy rainfall over 24 and 48 hours (precipitation accumulation of 60 mm and 80 mm, respectively) on the S1-SSM values was analyzed as a function of the time lag between the rainfall event and the S1 acquisition. The results show that similar soil moisture conditions could be obtained on the S1-SSM maps that are acquired after an intensive rainfall event a couple of days prior to the S1-SSM maps, and the S1-SSM maps acquired after light rain (few mm) 24 hours before the S1-SSM maps. Finally, we studied the effect of the temporal resolution of satellite images on detecting heavy rainfall events using the SMAP soil moisture product at one-day temporal resolution. The results show that despite the difference in soil moisture values in SMAP data after heavy and light rainfall events, the one-day revisit time doesn’t also allow an easier detection of heavy rainfall events. Therefore, the detection of heavy rainfall is not always obvious, whether using the S1-SSM maps with a revisit period of six days (at plot scale) or the SMAP-SSM maps with a very high temporal resolution of one day (at 9 km × 9 km) since many environmental parameters (evaporation, soil texture…) could also affect the value of SSM.
